# Longitudinal study of socio-emotional cognitive processing in individuals with anorexia nervosa and the impact of autistic characteristics on neural processing

**DOI:** 10.3389/fpsyg.2025.1583417

**Published:** 2025-06-23

**Authors:** Daniel Halls, Jenni Leppanen, Steve Williams, Kate Tchanturia

**Affiliations:** ^1^Department of Psychological Medicine, King’s College London, Institute of Psychiatry, Psychology and Neuroscience, London, United Kingdom; ^2^Sir Peter Mansfield Imaging Centre, School of Medicine, University of Nottingham, Nottingham, United Kingdom; ^3^Department of Neuroimaging, Institute of Psychology, Psychiatry and Neuroscience, King’s College London, London, United Kingdom; ^4^South London and Maudsley NHS Foundation Trust National Eating Disorder Service, London, United Kingdom; ^5^Department of Psychology, Illia State University, Tbilisi, Georgia

**Keywords:** anorexia nervosa, autism, socio-emotional and cognition, longitudinal, fMRI

## Abstract

**Background:**

Difficulties in socio-emotional cognitive processing are a key feature in individuals with anorexia nervosa (AN); however, the underlying neural processing, particularly longitudinal, is poorly understood. Compounding difficulties is the presence of overrepresented autistic characteristics, and it is unclear how these impact socio-emotional cognitive neural processing in individuals with AN.

**Method:**

A total of 92 participants, including 65 individuals with AN and 27 controls, took part in a longitudinal assessment at two time points, approximately 2 years apart, by undertaking socio-emotional cognitive tasks while undergoing functional magnetic resonance imaging (fMRI). A multivariate approach was used to predict autistic characteristics from generated maps from the AN group.

**Results:**

A group-by-time interaction effect was demonstrated in several brain regions in response to tasks, with the regions with the strongest evidence being the right frontal operculum/pole. The multivariate approach revealed a wide distribution of brain regions contributing to autistic characteristics.

**Conclusion:**

Neural changes over time in the right frontal operculum/pole potentially represent a compensatory mechanism for cognitive difficulties. Autistic characteristics in individuals with AN are instantiated and impact a wide distribution of neural regions, particularly during socio-emotional cognitive processing.

## 1 Introduction

Anorexia nervosa (AN) is a serious eating disorder (ED), defined by restriction of dietary intake, intense fear of weight gain, and disturbance of body perception (American Psychiatric Association, 2013). Theoretical models of AN have highlighted that different socio-emotional cognitive processing styles are important features in AN, thought to be causative and maintaining mechanisms, as well as acting as a barrier to treatment (Treasure and Schmidt, 2013). These models postulate that socio-emotional difficulties cause the internalisation of ED values, while cognitive processing styles produce rigid dieting behaviour, resulting in exacerbation of socio-emotional cognitive processing difficulties and illness state (Treasure and Schmidt, 2013). Support for this theoretical framework and its implications comes from a wide range of behavioural work exploring socio-emotional cognition in individuals with AN (Harrison et al., 2012; Sander et al., 2021; Sala et al., 2023). Previous evidence has shown that emotional disorder traits and interpersonal/social difficulties are associated with increased severity and relapse of ED symptoms in individuals with AN (Cardi et al., 2018; Sander et al., 2021; Sala et al., 2023; Di Lodovico et al., 2023). Previous research has also shown that individuals with AN show a variety of differing cognitive profiles compared to healthy controls (HC), including weak central coherence (CC; Harrison et al., 2012; Lang et al., 2016; Amlung et al., 2019), with differences in CC having the largest effect size than other cognitive domains (Stedal et al., 2021). Deficits in CC (the ability to process information in context) are believed to contribute to illness state by preventing individuals with AN from comprehending the larger picture related to illness state and treatment (Treasure and Schmidt, 2013; Harrison et al., 2012; Lang et al., 2016).

Despite the consensus from behavioural evidence supporting the importance of socio-emotional cognition in individuals with AN, how these characteristics are instantiated on a neural level is poorly understood. Findings examining socio-emotion cross-sectionally have implicated atypical functioning in a range of regions, including the fusiform, insula, and amygdala (Fonville et al., 2013a; Bang et al., 2016; Leppanen et al., 2017). However, previous research, including our previous study, has reported no whole-brain exploratory between-group differences (Halls et al., 2021). Findings exploring CC are lacking, with limited cross-sectional work implicating atypical function in the precuneus and fusiform gyrus (Fonville et al., 2013b). As our previous study reported, there were no group differences in a CC task (Leslie et al., 2021). Further compounding the confusion about the neural correlates of CC is work exploring within-AN-group correlations between areas activated in a CC task and treatment response, with no significant correlations being found (Garrett et al., 2014).

However, examining longitudinal fMRI research suggests differing responses over time may be a key neural correlate of cognitive processing (Decker et al., 2015; Castro-Fornieles et al., 2019; Doose et al., 2020). Decker et al. (2015) found atypical functioning in the cingulostriatal circuit and excessive cognitive control in individuals with AN, which normalised over time during treatment, with the authors arguing this normalisation is needed for individuals with AN to make non-ED value judgements. Castro-Fornieles et al. (2019) found similar results using a set-shifting task, with individuals with AN showing atypical functioning in a variety of regions including the cerebellum, which normalised over time. The authors argue that this could be used as a biomarker of the illness state. Finally, Doose et al. (2020) found reduced activation in the default mode network over time, which they argued may reflect the relaxation of cognitive control in individuals with AN. However, issues in previous longitudinal work make it difficult to make interpretations regarding socio-emotional cognition in individuals with AN. Socio-emotional processing and CC are underexplored longitudinally, with previous work favouring exploring increased cognitive control instead. Additionally, methodological issues such as a lack of a follow-up group HC (Doose et al., 2020) do raise difficulties in interpreting previous work. If, as previous work suggests, neural and behavioural functioning are temporally fluid and not fixed (Decker et al., 2015), further exploratory longitudinal work is needed.

Another confounding issue when exploring socio-emotional cognitive processing is the impact of autistic characteristics*. Previous studies have shown that autistic characteristics are overrepresented in individuals with AN (Kerr-Gaffney et al., 2020; Kinnaird and Tchanturia, 2021; Kerr-Gaffney et al., 2021; Tchanturia, 2022). However, importantly, autistic individuals and individuals with AN share similar socio-emotional cognitive profiles, including interpersonal difficulties and weak CC (Kinnaird and Tchanturia, 2021; Kerr-Gaffney et al., 2021). These shared socio-emotional cognitive processing styles are postulated to play a key mediating role in the relationship between autistic traits and ED traits (Kerr-Gaffney et al., 2020; Leppanen et al., 2022). For example, autistic traits have been shown to predict future socio-emotional difficulties (Leppanen et al., 2022), and social anxiety is a bridging symptom between ED and autistic traits in individuals with AN (Kerr-Gaffney et al., 2020). Autistic individuals have been found to have weak CC when viewing social scenes (Tassini et al., 2022), and weak CC is a common mechanism between ED and autistic individuals (Lang et al., 2016).

The relationship between autistic characteristics and neural processing in individuals with AN is poorly understood, as little work has been done in this area. In our previous study, we found that autistic characteristics in individuals with AN, while undergoing a theory of mind task, are correlated to blood oxygen level-dependent (BOLD) response within the right extrastriate cortex (Leslie et al., 2020). However, it is difficult to interpret this finding due to the absence of other similar studies exploring the role of autistic features, as well as the problems of informal reverse inferencing of behaviour from brain maps using univariate fMRI methods (Poldrack, 2011). A potential solution for attempting to establish a brain behaviour connection, in the absence of previous research, is multivariate pattern analysis (MVPA) decoding. MVPA decoding predicts behaviour from brain maps and can form a basis for formal reverse inferencing (Poldrack, 2011; Hoyos-Idrobo et al., 2018). MVPA decoders also convey neurobiological information that univariate methods may find difficult to do, such as identifying neural patterns that drive predictions of behaviour (Hoyos-Idrobo et al., 2018). In individuals with AN, MVPA has been used successfully to obtain the neural representation of anxiety traits and to predict treatment outcomes (Boehm et al., 2021; Seiger et al., 2023). In autistic individuals, MVPA has been used to predict the severity of autistic characteristics (Liu and Huang, 2020). Arguably, then, MVPA decoding may be better suited than univariate methods in exploring the relationship between autistic features and neural processing of socio-emotional cognition in individuals with AN.

To explore socio-emotional cognitive processing and the impact of autism, this study had two aims. The first aim was to longitudinally explore the neural processing of socio-emotional cognitive processing in individuals with AN. This was done by having a sample of individuals with AN and HC, at two time points, approximately 2 years apart, undergo a series of socio-emotional cognitive tasks while undertaking fMRI scanning. Due to a lack of specific task-based longitudinal work conducted in individuals with AN, an exploratory whole-brain approach was used rather than examining regions of interest. To fully explore this aim, we also conducted within AN group correlation analysis to explore the relationship between symptomology and neural processing. The second aim was to explore the relationship between autistic characteristics and neural processing of socio-emotional cognition in individuals with AN. This was done by using MVPA decoders to predict autistic characteristics from socio-emotional processing tasks in our sample.

## 2 Methods

### 2.1 Participants

The present study is a longitudinal continuation of our previous cross-sectional work (Halls et al., 2021; Leslie et al., 2021). A total of 92 participants, 65 individuals with AN and 27 HC, took part in two MRI sessions, time point 1 (TP1, June 2017–February 2019) and time point 2 (TP2, October 2019–March 2022). A total of 40 individuals with AN from our sample underwent the Autism Diagnostic Observation Schedule second edition (ADOS-2) at TP1 to assess our study’s second aim of exploring autistic characteristics and the neural processing of socio-emotional cognition in individuals with AN. All participants were female, aged between 12 and 27 years at the time of enrolment, and did not have any neurological impairment (e.g., epilepsy), serious brain injury, or intellectual disability. Before enrolment, all participants underwent the Structured Clinical Interview for DSM-5—research version (First et al., 2015). Individuals with AN were eligible to take part if they had a diagnosis of AN, as defined by the DSM-5 criteria, and were all at various illness stages, such as being acutely underweight and recovering. Participants with AN were recruited from clinical services, including the South London and Maudsley specialist ED Service, South-West London, and St George’s ED Service, as well as the UK ED charity Beat. HC participants were eligible if they had no history of ED and were recruited from King’s College London and the local community. All participants gave written informed consent at both time points, unless the participant was under 18 years of age, in which case, a legal caretaker provided consent. The study was approved by the London-Surrey National Research Ethics Committee (17/LO/2071 and 19/SC/0367). All procedures comply with the ethical standards of the Helsinki Declaration of 1975, as revised in 2008. For further information, please see Halls et al. (2021) and Leslie et al. (2021).

### 2.2 Measures

#### 2.2.1 Clinical and behavioural measures

Before the scanning session, participants at both time points completed the Eating Disorders Examination-Questionnaire (EDE-Q; Fairburn et al., 2009) and the Hospital Anxiety and Depression Scale (HADS; Zigmond and Snaith, 1983). Items exhibited acceptable internal consistency (Cronbach alpha = 0.748). Participants also completed a demographic questionnaire that included information on height and weight so that body mass index (BMI), defined as kg/m^2^, could be calculated. These items were collected so that the relationship between neural processing and symptomology could be fully explored, as well as providing clinical parameters of our population. Participants with AN at TP1 underwent the ADOS-2 to assess for autistic characteristics by a trained ADOS-2 administrator. The ADOS-2 is a gold standard clinical interview measure of autistic traits, which is appropriate in individuals with AN (Sedgewick et al., 2019). The interaction, communication, creativity, and stereotyped and repetitive domains of the ADOS-2 were used as a measure of autistic characteristics within our population. In total, 40 AN participants underwent the ADOS-2 assessment; however, one participant was removed due to being an extreme outlier, leaving 39 participants.

#### 2.2.2 fMRI tasks

The MRI scanning session consisted of two implicit emotion tasks, one exploring responses to happy and the second exploring responses to fearful emotions. The implicit emotional processing tasks consisted of showing 20 faces with 100% emotion, 20 partially emotional faces with 50% emotion, 50% neutral facial expression, 20 neutral expression faces, and 13 fixations cross. Faces were presented for 2 s with a 7 s interstimulus fixed to a Poisson distribution. Participants were asked to indicate whether faces were male or female to render emotional reactivity implicit. See Halls et al. (2021) for further details.

Following the implicit processing tasks, participants completed an embedded figures task (EFT) to test for differences in CC. In each trial, participants were presented with a target geometric shape and two complex shapes. Participants indicated which of the complex shapes, either on the left or the right of the screen, contained the target geometric shape. Trials were defined as either simple or complex, depending on the complexity of the shapes being searched. A central fixation cross was presented at the beginning, middle, and end of the task for 30 s, and the task lasted 450 s. See Leslie et al. (2021) for further details.

### 2.3 Procedure

At TP1, each participant undertook a screening call for eligibility for enrolment in the study. If the participant was eligible, they attended two study sessions. During the first session, participants underwent the ADOS-2 clinical interview and completed the clinical questionnaires. Participants were also screened for MRI safety. At TP2, as this was during the COVID-19 pandemic, this session took place online; however, no ADOS-2 was conducted. The second session was the same across both time points, with participants attending the Centre for Neuroimaging Sciences at King’s College London for an MRI scan. Each participant was tested around the same time (16:00–18:00). This scan consisted of a T1-weighted sequence, followed by the implicit emotional processing tasks and the EFT task. The order of tasks did not vary between time points or participants, such that all participants underwent the emotional processing task with happy stimuli, followed by fearful stimuli, and, finally, the EFT (please see Halls et al., 2021; Leslie et al., 2020 for further details).

### 2.4 Scan acquisition

Images were acquired at the Centre for Neuroimaging Sciences at King’s College London on a 3 T GE MRI scanner. A T1-weighted image was taken with the following parameters: echo time of 3.02 s, repetition time of 7.31 s, flip angle of 11 degrees, field of view of 270 mm, 256 × 256-pixel matrix, and slice thickness of 1.2 mm. Three T2* images depicting the BOLD response to the happy and fear implicit processing along with the EFT were acquired. All three T2* images had the following parameters: a 2 s repetition time, slice thickness of 3 mm, slice thickness of 3.3 mm, field of view of 240 mm, 64 × 64-pixel matrix, and a flip-angle of 75 degrees. The T2* images depicting BOLD responses to implicit emotional processing contained 183 time points, while the T2* image depicting the BOLD response to EFT contained 225 time points.

### 2.5 Statistical analysis

#### 2.5.1 Strength of evidence

The use of *p*-values alone to decide on the “significance” of results has been widely criticised (Wasserstein and Lazar, 2016; Benjamin and Berger, 2019), and crucially, *p*-values cannot decide on the strength of evidence (Benjamin and Berger, 2019). To ascertain the strength of evidence and significance in a robust manner, this study has implemented a combined Bayesian approach, along with the following recommendations taken from Benjamin and Berger (2019). *p*-values between 0.05 and 0.005 are called suggestive rather than significant. The Bayes Factor Bound (BFB), a function of the *p*-value representing the upper limit of where the Bayes factor lies, was calculated (Benjamin and Berger, 2019). From BFB, the probability of the null hypotheses was calculated and presented along with *p*-values and other model parameters to aid in data interpretation.

#### 2.5.2 Behavioural data

Differences between groups, time points, and a group-by-time interaction for the EDE-Q global score, HADS anxiety and depression scores, age, and BMI were explored using linear mixed effects models (LMEM), with participants as the random effects. All analyses were conducted in Python 3.10. For the task behavioural data, all variables were examined using Mann–Whitney U-tests, with family-wise error (FWE) control of the *p*-value using the Holm-Sidak method, applied due to the number of tests. BFB was calculated from uncorrected *p*-values. This study specifically examined group differences across both time points in the following variables: reaction time (RT) in selecting the gender of the emotional faces and the number of correctly identified faces in implicit emotion tasks. As well as RT in selecting a figure and the number of correct responses in the embedded figures task.

#### 2.5.3 Missing data

One participant had a single missing question on the EDE-Q, which was deemed missing at random. This missing data was imputed using a weighted average of K number of closest values, measured by Euclidean distance (Troyanskaya et al., 2001).

#### 2.5.4 MRI quality control and pre-processing

Quality control of MRI data was done by visually inspecting the output generated by MRIQC, a quality control pipeline (Esteban et al., 2017). Pre-processing was conducted using the standardised pre-processing pipeline fMRIPrep (Esteban et al., 2019, 2020), with no additional spatial smoothing (see Supplementary material section 1 for further details).

#### 2.5.5 MRI data analysis

First level modelling was conducted in SPM12^1^ using the Nipype Python framework. A general linear model was fitted to all tasks (implicit emotions and the EFT). BOLD denoising used a data-driven approach by including in the model the number of anatomical component corrections (aCompCors), which explains 50% of the variance, along with the 18 movement regressors of derivatives, squares, and squares of the derivatives of motion regressors. This denoising strategy has been shown to be superior to other denoising methods (Mascali et al., 2021). The time series was high-pass filtered at 120 s, then convolved with the canonical haemodynamic response function, as well as with its temporal and dispersion derivatives. To accommodate BOLD habituation in response to viewing repeated emotional faces, a parametric modulator of time was fitted to the implicit emotional processing task. Reaction time was added as a parametric modulator to the EFT to allow for potential differences in BOLD responses in trials where participants respond more quickly. A parametric modulator of numbers correctly guessing the figure was also generated but not included due to exhibiting a significant correlation with the time of trial. Temporal autocorrelation was modelled using SPM’s alternative pre-whitening strategy “FAST.” F-contrasts of the canonical haemodynamic response function, its derivates, and the parametric modulator were generated. For the implicit emotion tasks, the F-contrast was a linear contrast of neutral–partial emotion–full emotion, while EFT simple trials were contrasted against (>) complex trials (complex>simple).

To test our first aim, second-level analysis was conducted using two permutated models in Permutation Analysis of Linear Models (PALM; Winkler et al., 2014). The first model was a linear regression that explored the effect of time and a group-by-time interaction with random intercepts for participants, while the second model was a two-sample *T*-test to explore group differences. For the linear regression model, multi-level exchangeability blocks were defined, one permitting between-subjects shuffling and the other allowing permutations within-subjects. For the two-sample *T*-test model, the average within-subject response from both time points was modelled, and then permutated to explore group differences. For both models, to speed up permutations, only the tail of distribution was modelled, allowing a reduced number of permutations to be used (Winkler et al., 2016), in this case, 1,000. Thresholding of images was done using threshold-free cluster enhancement with a *p*-value set at 0.05, FWE-corrected.

To explore potentially significant clusters, contrast estimates from clusters were extracted and plotted, along with contrast means and standard deviations (SDs). To further explore effect sizes within clusters, LMEMs with contrast estimates as the dependent variable and group, time, and group-by-time interaction as independent variables were conducted. As the second-level PALM analysis had already suggested the potential significance of these clusters, only regression coefficients were examined. To further explore our first aim of longitudinal neural processing of socio-emotional cognitive processing in individuals with AN, contrast estimates from clusters were correlated using Spearman’s rank-order correlation to change scores of EDE-Q global, BMI, age, HADS anxiety, and HADS depression within the AN group. Correlation analysis was used over LMEM, as the LMEM cluster and behavioural measures models displayed severely skewed residuals. Due to the number of correlations being performed, *p*-values were FWE-corrected, and BFBs were calculated from uncorrected *p*-values. Finally, to further address the strength of evidence, the upper bound of the posterior odds (PO) and posterior probability (POST PB) of each potentially significant cluster was calculated by multiplying the prior odds of the cluster being activated by the task, with the BFB (Benjamin and Berger, 2019). The PO and POST PB give the updated odds/probabilities given the evidence that cluster being activated in response to a task (not the probability of a group difference), allowing for the strength of evidence to be assessed (Benjamin and Berger, 2019). The prior probability for each cluster was obtained from neurosythn^2^, an automated database of activation coordinate data, and then converted to odds. For clusters related to implicit emotional processing, prior odds from neurosythn’s emotional faces map were used, while for clusters related to the EFT, prior odds of activation for cognition were used.

To examine our second aim, the relationship between autistic characteristics and neural processing in AN, two MVPA regression decoders were used. The first decoder was Spacenet, a structured sparse method decoder with a total variation L1 regularisation prior (Dohmatob et al., 2015). The second was the fast regularised ensemble of models (FREM), a non-structured sparse method decoder using a support vector regression (Hoyos-Idrobo et al., 2018). These decoders have been shown to produce stable, informative weights and are appropriate for non-smoothed data (Janoos et al., 2010; Hoyos-Idrobo et al., 2018). The rationale for two decoders was to examine which model, the spatially sparse or more distributed model, can better predict autistic characteristics, therefore giving a better understanding of the underlying neurobiology.

The MVPA approach consisted of taking the mean response from the 1st level F-contrasts across both time points and all tasks from the 39 AN subjects who undertook the ADOS-2. These generated averaged socio-emotional cognitive processing contrast maps and were used as predictors for the decoder models. Domains of the ADOS-2 were used as dependent variables in the models. All models were trained on a training dataset consisting of 31 randomly selected AN participants, with models being tested by calculating *R*^2^ and mean absolute error on a held-out test dataset consisting of eight participants. Weights from models that did not perform arbitrarily worse (i.e., those with positive *R*^2^) were explored and converted to t-scores with *p*-values. BRB and hypothesis probabilities were then calculated to explore the significance of weights. Finally, model parameters, using the location of clusters from the whole-brain exploratory analysis, were extracted from models that did not perform arbitrarily poorly to explore if the whole-brain clusters were also predictors of autistic characteristics.

## 3 Results

### 3.1 Behavioural data

The average follow-up for individuals with AN was 2 years and 9.528 months (*7.488 months SD*), while for HC was 3 years and 1.188 months (*8.928 months SD*). No group difference was identified in the follow-up period (*p = 0.06, U-val = 1097.500, CLES = 0.635, BFB = 2.183*). Results from the LMEM showed group differences in anxiety, depression, and EDE-Q global scores, with individuals with AN having increased scores compared to HC. AN individuals had decreased BMI compared with HC. A group-by-time interaction effect for EDE-Q existed, where, compared to HC, individuals with AN had decreasing EDE-Q scores between TP1 and TP2, while the HC group had marginally increased scores. No differences in the group-by-time interaction parameter were found for BMI or HADS. No differences between time points were detected for both domains of the HADS and the EDE-Q (see Table 1 for more details).

**Table 1:** Participants’ clinical measures presented by group and time point, as well as the results from linear mixed effects models exploring group, time, and group-by-time interaction differences.

BMI mean and STD				
	AN TP1	AN TP2	HC TP1	HC TP2
Mean (std)	18.096 (2.7112)	19.433 (3.299)	22.720 (3.248)	24.664 (4.923)

BMI mixed model				
	Coefficient	Std error	Significance metrics	95% CI
Group [AN]	−0.093	0.017	*p* = <0.001 BFB = > 1,000 Null % = <0.001	[−0.126–0.060]
Time [TP2]	0.053	0.018	*p* = 0.052 BFB = 2.41 Null % = 29.35	[0.016–0.089]
Interaction [AN TP2]	−0.024	0.022	*p* = 0.817 BFB = −2.225 Null % = 68.994	[−0.067–0.019]

HADS anxiety mean and STD				
	AN TP1	AN TP2	HC TP1	HC TP2
Mean (std)	10.371 (3.563)	11.785 (4.185)	4.962 (3.268)	6.192 (3.335)

HADS anxiety mixed effects model				
	Coefficient	Std error	Significance metrics	95% CI
Group [AN]	5.409	0.876	*p* = <0.001 BFB = > 1,000 Null % = <0.001	[3.692–7.126]
Time [TP2]	1.048	0.919	*p* = 0.175 BFB = 1.207 Null % = 45.307	[−0.753–2.850]
Interaction [AN TP2]	0.399	1.090	*p* = 0.198 BFB = −2.552 Null % = 71.850	[−1.738–2.535]

HADS depression means and STD				
	AN TP1	AN TP2	HC TP1	HC TP2
Mean (std)	7.645 (3.790)	6.815 (4.326)	3.000 (2.800)	2.962 (2.900)

HADS depression mixed effects model				
	Coefficient	Std error	Significance metrics	95% CI
Group [AN]	4.457	0.860	*p* = <0.001 BFB = > 1,000 Null % = <0.001	[2.772–6.142]
Time [TP2]	0.000	0.807	*p* = 0.992 BFB = −48.498 Null % = 98.000	[−1.584–1.583]
Interaction [AN TP2]	−0.704	0.955	*p* = 0.461 BFB = −1.030 Null % = 50.749	[−2.580–1.173]

EDE-Q global mean and STD				
	AN TP1	AN TP2	HC TP1	HC TP2
Mean (std)	3.258 (1.629)	2.551 (1.530)	0.832 (1.124)	0.938 (0.699)

EDE-Q global mixed effects model				
	Coefficient	Std error	Significance metrics	95% CI
Group [AN]	2.419	0.356	*p* = <0.001 BFB = > 1,000 Null % = <0.001	[1.722–3.116]
Time [TP2]	1.073	0.423	*p* = 0.724 BFB = −1.572 Null % = 61.113%	[0.243–1.903]
Interaction [AN TP2]	−2.205	0.500	*p* = 0.023 BFB = 4.244 Null % = 19.069	[−3.185–1.226]

Age mean and STD				
	AN TP1	AN TP2	HC TP1	HC TP2
Mean (std)	18.879 (3.489)	21.673 (3.509)	18.815 (2.936)	21.915 (3.132)

Age mixed effects model				
	Coefficient	Std error	Significance metrics	95% CI
Group [AN]	0.064	0.771	*p* = 0.999 BFB = −624.312 Null % = 99.84%	[−1.511–1.512]
Time [TP2]	3.100	0.130	*p* = <0.001 BFB = > 1,000 Null % = <0.001%	[2.845–3.354]

ADOS-2 domains means and STD for AN group				
	Interaction	Creativity	Communication	Stereotype
Mean (std)	1.825 (2.123)	0.450 (0.552)	1.375 (1.547)	1.850 (1.388)

AN, Anorexia Nervosa; ADOS-2, Autism Diagnostic Observation Schedule, second edition; CI, confidence interval; HC, Healthy Controls; TP2, Time point two; std, standard deviation; std error, standard error.

No group differences across time points were detected for RT for all fMRI tasks or for correctly selecting the embedded figure in the EFT or the face in the implicit emotion task (see Supplementary material one section two for more details).

### 3.2 Implicit emotional processing task

**Figure 1:**
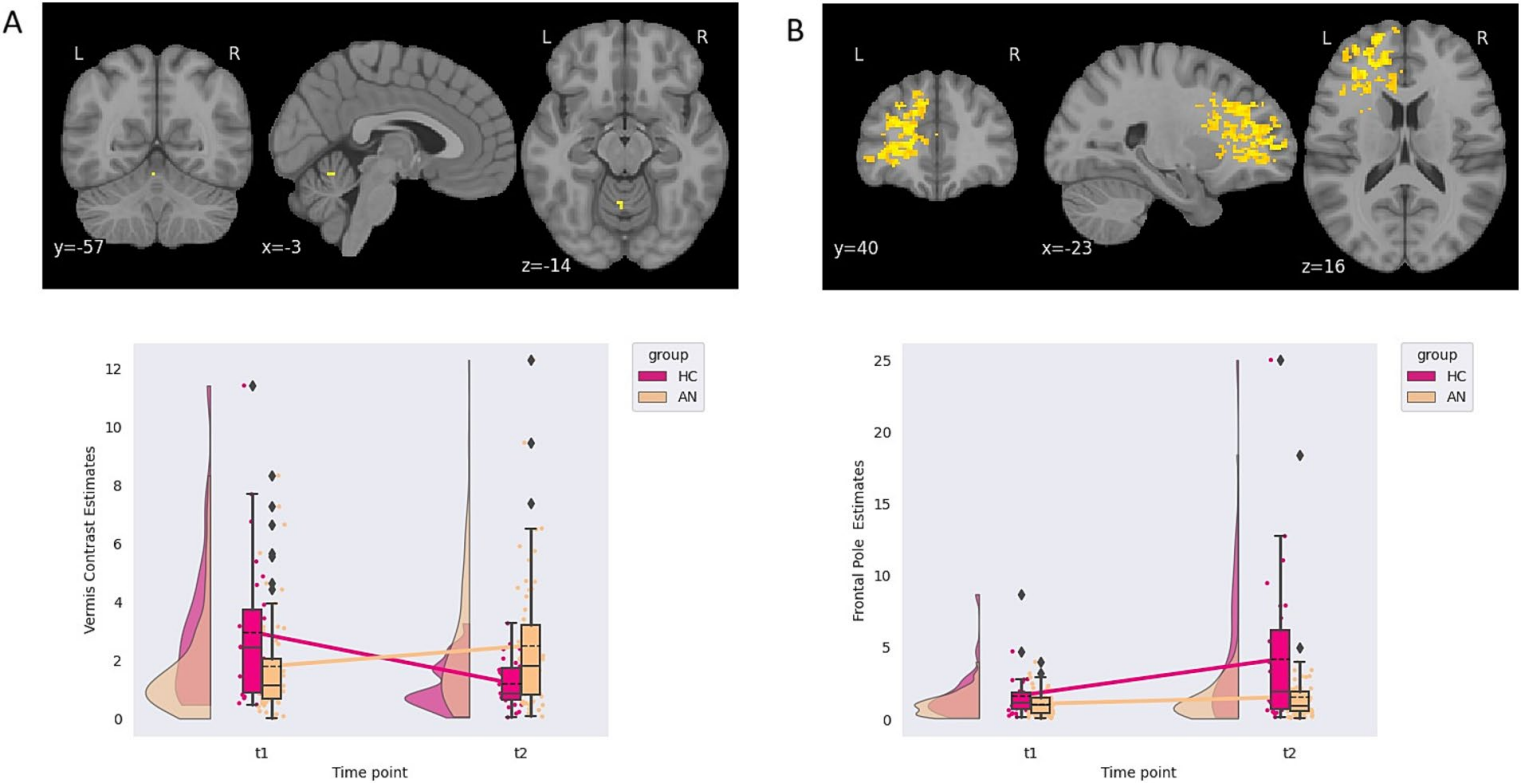
**(A)** Contrast estimates demonstrating a group-by-time interaction from the happy implicit emotional processing task. **(B)** Contrast estimates demonstrating an effect of time from the fear implicit emotional processing task.

A single group-by-time interaction for the F-contrast of the linear increase from neutral to happy faces (100% neutral to 50% happy to 100% happy), located in the left vermis, was found. Plotting of contrast estimates and examining the LMEM demonstrated that AN had increasing, while HC had decreasing, contrast estimates between time points (see Table 2; Figure 1 for further details). Therefore, this result showed that individuals with AN had an increasing response over time in the left vermis compared with HC. However, the PO and POST PB of these findings were 0 and 0%, respectively, due to the prior odds of this region being activated for emotional faces set at 0. No group or time differences were found for the happy faces contrast. Correlating contrast values from the vermis cluster to changing EDE-Q, anxiety, depression, age, and BMI showed no correlations above 0.2 or *p*-values less than 0.05 (see Supplementary material one section three).

**Table 2:** Cluster values for the implicit emotion task.

Cluster	Peak co-ordinates (MNI)	Size mm^3^	Significance metrics	Location	Contrast estimates (SD)	Coefficients from LMEM
Happy interaction cluster	X: −4.5 Y: −56.5 Z: −14.5	32	*p* = 0.04 BFB = 2.79 Null % = 26.35 Prior PB = 0.0 Post O = 0.0 Post PB = 0.0	L Vermis* L Cerebellum*	AN TP1: 1.81 (1.77) HC TP1: 2.97 (2.55) AN TP2: 2.51 (2.33) HC TP2: 1.22 (0.83)	AN[TP2]: 2.45 (0.62)
Fear time cluster	X: −18.5 Y: 43.5 Z: 19.5	28,080	*p* = 0.01 BFB = 6.69 Null % = 13.0 Prior PB = 0.0 Post O = 0.0 Post PB = 0.0	L Frontal pole** L Middle Frontal gyrus** L Paracingulate gyrus** L Superior Frontal gyrus**	AN TP1: 1.06 (0.81) HC TP1: 1.65 (1.74) AN TP2: 1.54 (2.39) HC TP2: 4.17 (5.51)	AN [TP2]: 2.53 (0.71)

* AAL atlas.

** Harvard Oxford atlas.

AN, Anorexia Nervosa; BFB, Bayes Factor Bound; HC, Healthy controls; L, left; LMEM, linear mixed effect model; mm^3^, millimetres; MNI, Montreal Neurological Institute, Null %, probability of hypothesis; P, *p* value; Prior P, prior probability; Post O, posterior odds; Post PB, posterior probability; R, right; SD, Standard deviation; TP1, time point one; TP2, time point 2.

An effect of time, independent of the group, in response to a linear increase from neutral to fear faces (100% neutral to 50% fearful to 100% fearful) was found in the left frontal pole. No group or group by time interaction differences were found for the fearful faces. Again, the PO and POST PB of these findings were 0 and 0%, respectively, due to the prior odds of this region being activated with emotional faces being 0 (see Table 2; Figure 1 for further details). A suggestive correlation between changing BMI scores and contrast values was found (*rho = 0.186, p = 0.035, BFB = 3.107*). The *p*-value did not survive multiple comparison correction.

### 3.3 Embedded figures

**Figure 2:**
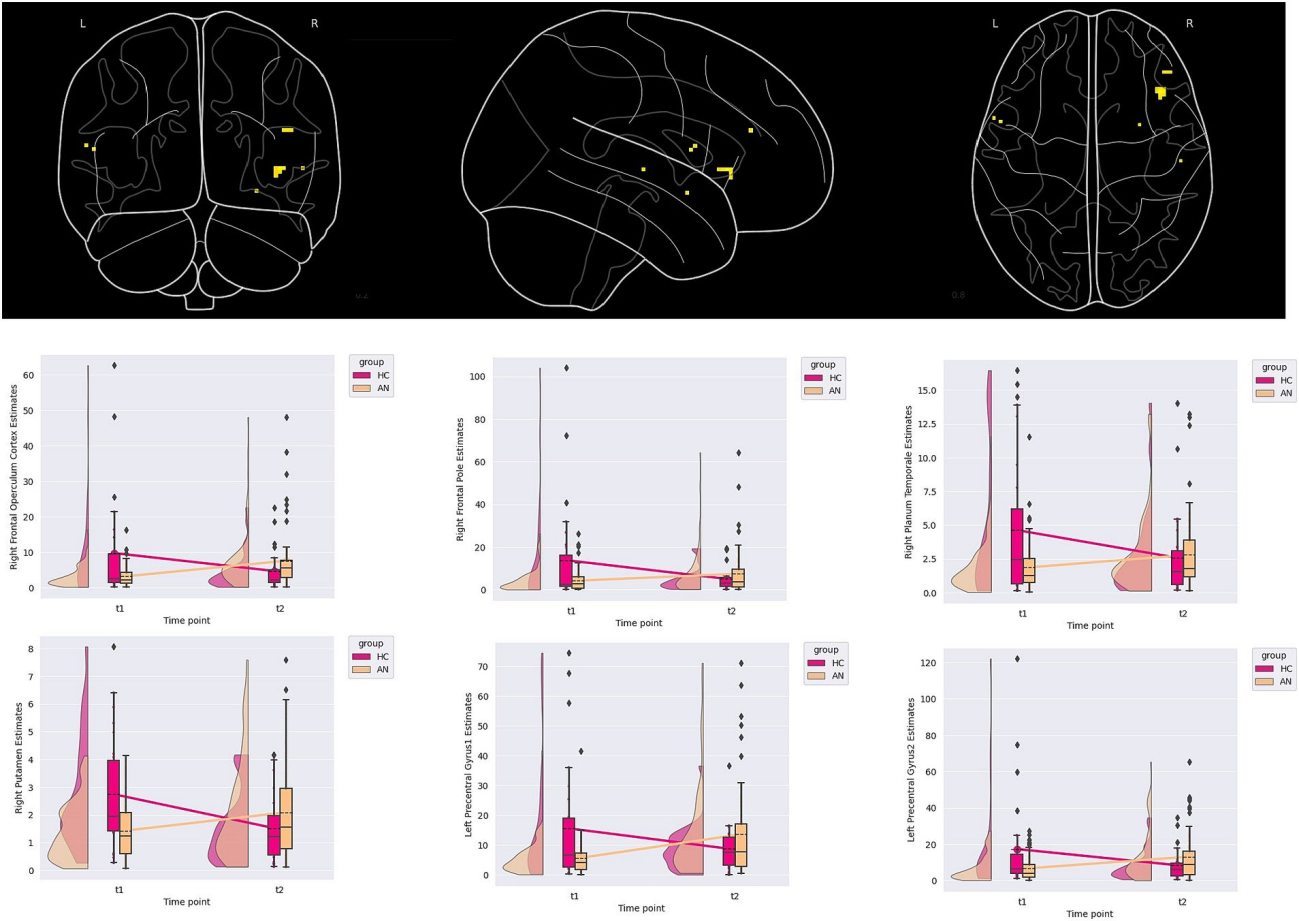
Contrast estimates demonstrating a group-by-time interaction from the embedded figure task.

A group-by-time effect in six clusters was demonstrated, two located in the left precentral gyrus and four right lateralised clusters located in the frontal operculum, frontal pole, planum temporale, and putamen. Examining the LMEM and cluster plots, all clusters demonstrated that HC had decreased, while the AN had increasing contrast estimates over time. The PO and POST PB for clusters ranged from 2.12 to 4.62 and 0.53 to 0.78, respectively (see Table 3; Figure 2 for details).

**Table 3:** Cluster values for the EFT task group-by-time interaction contrast.

Cluster	Peak co-ordinates (MNI)	Size mm^3^	Significance metrics	Location*	Contrast estimates (SD)	Coefficients from LMEM
Cluster 1	X: 41.5 Y: 25.5 Z: 1.5	88	*p* = 0.028 BFB = 3.66 Null % = 21.46 Prior PB = 0.49 Post O = 3.52 Post PB = 0.71	R Frontal Operculum R Frontal Orbital Cortex	AN TP1: 3.16 (2.96) HC TP1: 9.70 (14.88) AN TP2: 7.55 (8.75) HC TP2: 4.65 (5.58)	AN[TP2]: 9.45 (2.60)
Cluster 2	X: 45.5 Y: 37.5 Z: 21.5	24	*p* = 0.038 BFB = 2.95 Null % = 25.30 Prior PB = 0.61 Post O = 4.62 Post PB = 0.78	R Frontal Pole	AN TP1: 4.24 (5.29) HC TP1: 13.83 (24.38) AN TP2: 7.47 (10.99) HC TP2: 5.08 (5.80)	AN[TP2]: 11.97 (3.74)
Cluster 3	X: 53.5 Y: −16.5 Z: 1.5	8	*p* = 0.049 BFB = 2.49 Null % = 28.0 Prior PB = 0.46 Post O = 2.12 Post PB = 0.53	R Planum Temporale	AN TP1: 1.87 (1.70) HC TP1: 4.62 (5.39) AN TP2: 2.80 (2.85) HC TP2: 2.57 (3.24)	AN[TP2]: 2.98 (1.02)
Cluster 4	X: 29.5 Y: 5.5 Z: −10.5	8	P = 0.035 BFB = 3.12 Null % = 24.27 Prior PB = 0.47 Post O = 2.77 Post PB = 0.64	R Putamen	AN TP1: 1.43 (1.02) HC TP1: 2.74 (1.99) AN TP2: 2.10 (1.78) HC TP2: 1.52 (1.28)	AN[TP2]: 1.88 (0.49)
Cluster 5	X: −54.5 Y: 7.5 Z: 11.5	8	*p* = 0.032 BFB = 3.34 Null % = 23.03 Prior PB = 0.44 Post O = 2.63 Post PB = 0.62	L Precentral Gyrus	AN TP1: 5.70 (6.00) HC TP1: 15.61 (20.67) AN TP2: 13.61 (15.49) HC TP2: 8.76 (7.46)	AN[TP2]: 14.77 (4.20)
Cluster 6	X: −58.5 Y: 9.5 Z: 13.5	8	*p* = 0.037 BFB = 2.99 Null % = 25.03 Prior PB = 0.55 Post O = 3.66 Post PB = 0.73	L Precentral Gyrus	AN TP1: 6.74 (6.94) HC TP1: 17.32 (27.35) AN TP2: 13.11 (14.07) HC TP2: 8.42 (8.77)	AN[TP2]: 15.26 (4.41)

* Harvard Oxford atlas.

AN, Anorexia Nervosa; BFB, Bayes Factor Bound; HC, Healthy control; L, left; LMEM, linear mixed effect model; mm^3^, millimetres; MNI, Montreal Neurological Institute, Null %, probability of hypothesis; P, *p*-value; Prior PB, prior probability; Post O, posterior odds; Post PB, posterior probability; SD, Standard deviation; TP1, time point one; TP2, time point 2.

Several suggestive correlations between changes in anxiety scores, age, and depression scores within the AN group were found, though the *p*-value no longer suggested significance once corrected for multiple comparisons (see Supplementary material one section three). For the right frontal pole, a suggestive correlation with changes in depression scores was found (*rho = 0.216, p value = 0.018, BFB = 5.117*). For the right planum temporale, a correlation to aging was suggested (*rho = 0.187, p-value = 0.033, BFB = 3.231*). Finally, for the left pre-central gyrus cluster located at X = −54.5, Y = 7.5, and Z = 11.5, a suggestive correlation to changes in anxiety scores was found (*rho = 0.235, p-value = 0.010, BFB = 8.201*).

### 3.4 Autistic characteristics and socio-emotional cognitive processing

The FREM decoder was better able to predict the stereotyped and repetitive behaviours (*mean absolute error = 0.84, R*^2^ *= 0.18*) than the communication (*mean absolute error = 1.53, R*^2^ *= <0.01*) domains from contrast maps. The FREM decoder performed arbitrarily worse for the social interaction (*mean absolute error = 1.15, R*^2^ *= −5.11*) and the creativity (*mean squared error = 0.85, R*^2^ *= −3.18*) domains. The Spacenet decoder was best able to predict creativity (*mean absolute error = 0.48, R*^2^ *= 0.02*) but performed arbitrarily worse for the stereotyped and repetitive (*mean absolute error = 1.21, R*^2^ *= −0.47*), the social interaction (*mean absolute error = 0.98, R*^2^ *= −3.08*), and the communication (*mean absolute error = 1.52, R*^2^ *= −0.12*) domains.

Exploring the weights for the Spacenet decoder in the creativity domain model demonstrated no significant predictors, whereas the FREM stereotyped and repetitive behaviours and communication models revealed a wide spatially distributed number of significant predictors. None of the clusters from the whole-brain exploratory analysis were significant predictors of autistic characteristics (see Supplementary material one section four. For the full list of significant predictors, see Supplementary material two for the ADOS communication FREM model and Supplementary material three for ADOS stereotyped and repetitive behaviour FREM model).

## 4 Discussion

This study had two exploratory aims: the first aim was to examine longitudinally the neural basis of socio-emotional cognitive processing by using a whole-brain analysis. Our results implicated a group-by-time interaction in numerous regions when completing the EFT and happy implicit emotional processing task. An effect of time in a large frontal cluster was also implicated during the implicit processing of fearful faces. Our second aim was to explore the relationship between autistic characteristics and the neural processing of socio-emotional cognition in individuals with AN using a MVPA decoding paradigm. Our results suggested that the decoders could predict autistic characteristics related to communication, stereotyped, and repetitive behaviours, as well as creativity.

Our whole-brain exploratory analysis findings demonstrated a group-by-time effect, which was in the left vermis of the cerebellum for the happy implicit emotional processing task. To the best of our knowledge, this region has not been implicated in socio-emotional tasks before in individuals with AN, but has been in resting-state analysis (Amianto et al., 2013) and cognitive tasks (Kullmann et al., 2014). However, given that the POST PB of this region being activated with emotional faces is 0%, the left vermis is unlikely to reflect a neural correlate of socio-emotional processing. Possible alternative interpretations could be that this finding may be a false positive, given the weak evidence for this finding, or may reflect a differing visual-motor response in individuals with AN. Terms with the highest prior odds taken from neurosythn for this cluster are motor-related, and previous evidence has highlighted that individuals with AN do have a slower motor response, not performing as well in visual-motor skills tasks (Kjaersdam Telléus et al., 2015).

The other significant whole-brain exploratory analysis finding was a group-by-time effect in the right frontal pole, operculum, orbital cortex, right putamen, and left precentral gyrus when undertaking the EFT. The clusters located in the right frontal operculum and frontal pole exhibit the strongest evidence. Previous socio-emotional research has implicated the right inferior frontal gyri (which correlates to the right frontal operculum) in individuals with AN (McAdams et al., 2016; Westwater et al., 2021). However, previous work using detail-orientated cognitive tasks has implicated several other regions not found in this study, such as the ventrolateral prefrontal cortex (Garrett et al., 2014; Lao-Kaim et al., 2015). Despite this conflict, a possible neuropsychological mechanism for our findings could be that, as there were no group differences in CC task performance, increased responses in frontal regions over time may reflect a neural compensatory mechanism in individuals with AN, allowing them to overcome known weak CC difficulties. This may also be the case with the right putamen, as previous studies have implicated this region in an illness state during a cognitive control task (Kullmann et al., 2014; Eddy et al., 2023), though the evidence for the right putamen is weaker in our study.

The relationship between behavioural and clinical features and neural responses increasing is important to delineate. Changes in depression and anxiety scores, as well as aging, were positively correlated to contrast values within the AN group for three clusters, though they did not survive multiple comparison correction. Examining the strength of the evidence, the aging correlation is unlikely to reflect a true result due to the low BFB and rho value. However, examining the strength of evidence for changing depression and anxiety scores indicates moderate evidence for a correlation that, possibly due to low statistical power, could not survive correction for multiple comparisons. This is supported by previous behavioural research in ED demonstrating that depressive symptoms are strongly associated with cognition (Aloi et al., 2015). However, attempting to place these findings in the context of previous fMRI research exploring CC is difficult due to the lack of previous research. The research that has been done examining the neural response of CC has either found no group differences (Leslie et al., 2021), so no relationship could be explored, or did not explore relationships between activation and mood symptoms (Fonville et al., 2013b). Work examining cognition in general is also inconclusive as trait anxiety has been found to correlate with dorsolateral prefrontal cortex responses (Sultson et al., 2016), but no correlation between mood symptoms and neural responses has been reported (Castro-Fornieles et al., 2019). Clearly, further work exploring the link between anxiety and depression and neural activation in relation to CC needs to be done. Another explanation for the group-by-time effects seen could be different neurodevelopmental trajectories in individuals with AN and HCs. Neurodevelopment of the cognitive functions associated with frontal lobes has been shown to not fully develop until later in adulthood (Kolk and Rakic, 2021). As our participants were adolescents at TP1, neurodevelopment of cognition, as well as the underlying frontal neural architecture, is to be expected. Further support for this comes from previous research suggesting a developmental element in AN pathogenesis (Remberk et al., 2021), although clearly, further longitudinal work is needed before a case regarding neurodevelopment can be made.

Our second aim was to explore the relationship between autistic characteristics and neural processing of socio-emotional cognition in individuals with AN by using two MVPA decoders, the spatial sparse encoder Spacenet and the more distributed model FREM. Interpreting these findings is difficult due to the limited sample size used to train and test these models, and this alone may explain the findings. Therefore, results and interpretations should be treated with caution; however, a few interpretations can tentatively be made. Our findings suggest that autistic characteristics of communication and stereotyped and repetitive behaviours are instantiated by and impact a wide spatial distribution of regions on a neural level. Additionally, as no single brain region model weight contributed considerably more to the models, this suggests that these autistic characteristics impact a wide distribution of regions evenly in individuals with AN during socio-emotion cognitive processing. This hypothesis of autistic traits affecting a wide range of evenly spatially distributed regions is supported by work done with autistic individuals. Using MVPA to predict autistic traits in autistic individuals, large-scale resting state network alterations have been implicated, with a wide spatial distribution of regions equally contributing to the model (Liu and Huang, 2020). Task-based fMRI has also shown reduced global synchrony in autistic children while watching a film, which has been argued to reflect differences in social stimuli processing (Lyons et al., 2020). Another Interesting finding from our MVPA is that none of the whole-brain clusters indicating group-based differences were significant predictors in the autistic trait MVPA models. This suggests that on a neural level, there is a delineation of socio-emotional cognitive processing associated with ED characteristics and socio-emotional processing associated with autistic characteristics. For creativity autistic characteristics, a more spatially constrained model was a better fit than a wider distributed model, such that creativity autistic characteristics are instantiated by a more localised set of regions. However, due to a lack of significant predictors, it is difficult to ascertain if this is the case with creativity autistic characteristics, and studies with larger sample sizes are needed before confirming preliminary interpretations.

Our results also highlight several null results that deserve further exploration. Our main null result was that no group differences were seen in any of the tasks and the inability of the decoders to predict social interaction behaviours. One potential reason may be due to this study’s small sample size and inability to delineate groups based on illness stage, resulting in false negative findings. Due to the problems of having several small underpowered sub-groups that would question the validity of our results, this study did not delineate based on illness state, but rather to maintain power, which merged participants with AN into one group. However, future studies should try and delineate the illness stage. A possible reason why the decoders were unable to predict social interaction could be the tasks used in this study. None of the tasks used had an interaction element beyond looking at stereotyped faces, meaning that social interaction was not actually tested for and, therefore, potentially unable to be predicted. Finally, another possible explanation is that rather than the neural response demonstrating a group-based difference at any given temporal point, it is possible that evolving neural responses over time are the salient neurobiological mechanism underpinning socio-emotional cognitive difficulties in AN. This interpretation would fit with cross-sectional studies that found no whole-brain exploratory group-based differences in individuals with AN (Bang et al., 2016; Leppanen et al., 2017). If this is correct, then more longitudinal work rather than cross-sectional work is needed to explore changing neural responses in individuals with AN.

This study is not without its limitations. As mentioned above, this study could not delineate illness state or age due to sample size. The reason this study’s sample size was not as large was due to recruitment taking place during the COVID-19 pandemic, which greatly impacted the recruitment numbers. A larger sample size would have allowed for a delineation of the AN group by illness state and all groups by age. This inhomogeneity may explain unexpected findings, such as a clear lack of correlation between age and CC processing. Given that CC is a cognitive task and cognitive development in adolescence is related to networks involving the right inferior frontal gyrus (Palmer et al., 2025), a clear positive correlation was expected.

Another limitation is the missing ADOS-2 scores, as 33% of the sample did not have ADOS scores available. These scores were missing at random due to a variety of reasons, such as patients not wishing to take part in the ADOS-2 or not fully completing the ADOS-2 interview. This limitation may explain some of this study’s null MVPA findings, and a larger sample is needed to fully explore our secondary aim. A further limitation is missing information on what type of treatment modality participants with AN were receiving before, during, and between time points. This information was not available to us due to recruiting from a wide range of clinical services and a charity organisation (Beat). Finally, the choice of prior odds was taken from meta-analytical maps not specific to AN. It may be the case that the prior odds for certain cluster activations are higher or lower in AN, and this would affect our PO. However, information on odds/probabilities of activation is not routinely reported in the AN literature, despite recommendations to do so (Benjamin and Berger, 2019). If this became routine practice, more specific priors could be specified, thereby improving the strength of evidence in neuroimaging research in AN.

In conclusion, this study, by using novel statistical approaches, has been able to demonstrate that several regions, particularly the right frontal operculum, exhibit an evolving response in individuals with AN, possibly reflecting a compensatory mechanism linked to anxiety and depression. This study has also been able to elicit, using modern MVPA decoding and machine learning, that autistic characteristics are instantiated and affect a wide set of spatially distributed regions.

## Data Availability

The raw data supporting the conclusions of this article will be made available by the authors, without undue reservation.

## Footnotes

^1^ http://www.fil.ion.ucl.ac.uk/spm

^2^ https://neurosynth.org/

## Author’s note

This study will use identity first language when describing autism, as this is preferred by the autistic community (Kenny et al., 2016).
